# Speed, Innovation, and Trust: Guiding Scientific Publishing in the Age of Artificial Intelligence

**DOI:** 10.7759/cureus.108762

**Published:** 2026-05-13

**Authors:** Alexander Muacevic

**Affiliations:** 1 Neurosurgery and Radiosurgery, European Radiosurgery Center Munich, Munich, DEU

**Keywords:** artificial intelligence in medicine, artificial intelligence in scientific writing, artificial intelligence journal, artificial intelligence medical journals, scientific writing and artificial intelligence

## Abstract

Scientific publishing is changing - and it's changing fast. Digital platforms have made it easier than ever to share research across borders, open-access models have pulled down paywalls that once limited who could read or contribute to scientific discourse, and global collaboration has become the norm rather than the exception. Into this already shifting landscape, artificial intelligence (AI) has arrived - quietly at first, and now with considerable force - touching nearly every stage of how research gets done, analyzed, and communicated.

The promise here is real. But so is the tension it creates. The central question facing the scientific community isn't whether to embrace these changes - that ship has largely sailed - but whether we can move this quickly without eroding the credibility that makes science worth doing in the first place.

For journals, this isn't a theoretical problem. Editorial standards are the backbone of the scientific record, and right now, those standards are being stress-tested. As the Editor-in-Chief of Cureus, I think we're at a moment that calls for clarity, not hedging - a moment to say plainly what principles must hold even as everything else shifts.

Cureus was built around a straightforward idea that medical publishing was too slow, too exclusive, and too gatekept to serve science well. The journal set out to change that by reducing barriers to dissemination while keeping editorial rigor intact. That core mission hasn't changed. What has changed is the environment in which we pursue it. Two issues now sit at the center of that effort: the responsible use of AI in scholarly communication and the ongoing fight to protect research integrity.

## Editorial

Artificial intelligence (AI) as a tool for science

AI is now woven into the fabric of research in ways that would have seemed implausible a decade ago. Scientists use AI-assisted tools to cut through vast bodies of literature, make sense of datasets that would take humans years to analyze manually, and sharpen the clarity of their writing. On the publishing side, similar tools are being tested for everything from identifying suitable reviewers to screening manuscripts for red flags.

When these tools are used thoughtfully, they can genuinely improve the quality and efficiency of what journals do. Automated systems can detect manipulated images or flag statistical patterns that might signal fraud - tasks that are genuinely difficult to do at scale by hand. As submission volumes continue to climb across scientific disciplines, this kind of support isn't just helpful; it's probably necessary.

But generative AI also creates problems that didn't exist before. Large language models can write fluid, authoritative-sounding scientific prose while simultaneously inventing citations, distorting data interpretations, or presenting fabrications with complete confidence. When used without transparency, they quietly dissolve the intellectual accountability that authorship is supposed to represent.

This is precisely why organizations like the International Committee of Medical Journal Editors (ICMJE) and the Committee on Publication Ethics (COPE) have been clear on one point: AI systems do not qualify as authors and should not appear on manuscripts as such [1]. The logic isn't bureaucratic - it's about maintaining a chain of human responsibility for the claims that end up in the scientific record.

Cureus aligns with this position. AI is a tool, not a colleague. Authors remain responsible for what they submit, full stop.

In practice, this means three things for how we operate: (i) Disclosure of AI assistance: Any substantive use (such as for content or grammar) of generative AI tools - whether in drafting, analysis, or figure creation - must be declared by the authors. (ii) Human accountability for authorship: No AI system may appear as an author. All listed authors are accountable for the scientific validity of the work. (iii) Human-centered editorial judgment: AI-assisted screening may inform our process, but decisions rest with expert reviewers and editors.

The underlying principle is simple: technology should make publishing more transparent and trustworthy, not less.

Safeguarding research integrity

Whatever AI does or doesn't change, the core challenge of research integrity stays constant. Science depends on honest data, sound methods, and conclusions that follow from evidence. That has always been true, and it remains true now.

What has changed is the scale and speed of the problem. Across disciplines - and this journal is no exception - there's been a troubling rise in paper mills, fabricated datasets, manipulated peer review systems, and image fraud [2,3]. These aren't edge cases anymore. They represent a genuine structural vulnerability in the publishing ecosystem.

The lesson here is one we can't afford to ignore: the speed at which we disseminate research must never outpace our ability to verify it.

Protecting the record requires effort from everyone involved. Authors need to hold themselves to rigorous ethical and methodological standards. Reviewers and editors need to push back hard on sloppy study designs and opaque reporting. Publishers need systems capable of keeping up with evolving forms of misconduct.

Technology helps. AI-based detection tools for image manipulation, anomalous citation behavior, and statistical irregularities are getting better all the time. But there's no software substitute for a culture that values transparency and takes accountability seriously. That culture - or its absence - is what ultimately determines whether scientific literature can be trusted.

Role of journals in a changing landscape

Journals sit at a particular kind of crossroads right now. The tools and models of scientific communication are being renegotiated almost in real-time - AI, open science, evolving peer review structures - and journals have to make consequential decisions about how to respond without a clear roadmap.

The temptation in moments like this is to either dig in defensively or rush to adopt every new tool on the assumption that change equals progress. Neither instinct is quite right. What's needed is something harder: adapting to what's genuinely useful while holding on to the things that make scientific publishing worth anything in the first place - transparency, rigorous evaluation, and accountability that runs all the way through the process.

For journals built around open and accessible publishing, this tension is especially sharp. Lowering barriers to participation is a genuine good. But it stays good only if the work being published can be trusted. Access and quality aren't competing priorities; they must advance in tandem [4,5].

The path forward for Cureus

The founding idea behind Cureus was to challenge the assumptions baked into traditional medical publishing - to ask whether the barriers to dissemination were serving science or just protecting incumbents. That spirit of questioning hasn't dimmed. What's changed is the complexity of the environment we're navigating.

Going forward, three priorities will shape how we develop. First, we'll continue to bring responsible technological tools into the editorial process - not uncritically, but where they genuinely improve manuscript screening, support peer review, and make communication more efficient. Second, we'll put in place clear public policies on how AI may and may not be used in research and writing submitted to the journal, aligning with international editorial standards. Third, we'll reinforce our safeguards against misconduct, including stronger screening for plagiarism, image manipulation, and patterns that tend to signal organized publication fraud.

The broader publishing landscape will continue to evolve, whether journals are ready for it or not. The question isn't whether to change - it's whether to change thoughtfully. That distinction matters enormously. If the next era of scientific publishing is going to deliver on its potential, it needs to hold together two things that often pull apart: the speed and openness that make science more productive, and the scrutiny and accountability that make it worth trusting.

That balance is what Cureus is committed to. Ultimately, it's the only way to ensure that advances in how we publish research serve the actual purpose of science - generating knowledge, carefully and honestly, in the interest of human health.
